# Liver Abscess With a Markedly High Level of Carbohydrate Antigen 19-9

**DOI:** 10.4021/gr475w

**Published:** 2012-09-20

**Authors:** Yusuke Yoshino, Kazunori Seo, Ichiro Koga, Naohisa Matsunaga, Takatoshi Kitazawa, Yoriyuki Takamori, Yasuo Ota

**Affiliations:** a Department of Internal Medicine, Teikyo University School of Medicine, 2-11-1 Kaga, Itabashi-ku, Tokyo 173-8606, Japan

**Keywords:** Liver abscess, Carbohydrate antigen 19-9, Tumor marker

## Abstract

Serological tumor markers are useful for detection of malignancies and evaluation of disease progression. However, some markers are rarely elevated in patients with benign diseases and without malignancies. We herein present a case of a liver abscess with a highly elevated carbohydrate antigen (CA 19-9) level in both the serum and abscess fluid. The serological level of CA 19-9 decreased with treatment. Although CA 19-9 is known to be a specific tumor marker, high serum levels of CA 19-9 can be observed in patients with pyogenic liver abscesses. CA 19-9 may also be a marker for treatment response in patients with liver abscesses.

## Introduction

Many types of serological tumor markers have been investigated. They are useful for detection of malignancies and evaluation of disease progression. Tumor markers are specifically elevated in patients with malignancies [1]. Rarely, however, some markers may be elevated in patients with benign diseases and without malignancies. Detection of tumor markers can sometimes produce a false-positive result.

Carbohydrate antigen 19-9 (CA 19-9), a determinant of circulating oligosaccharide antigen, is a frequently used serologic tumor marker in the auxiliary diagnosis of gastrointestinal and biliary malignancies. Its epitope was identified by a monoclonal antibody that is reactive to a sialylated lacto-N-fucopentaose II carbohydrate epitope associated with glycoproteins and glycolipids on colorectal biliary carcinoma cells [2]. CA 19-9 is well known as a specific serological tumor marker, especially in pancreatic malignancies [3, 4].

We herein present a patient with a pyogenic liver abscess and a markedly elevated serum CA19-9 level, but no elevation of other tumor makers such as carcinoembryonic antigen (CEA), alpha-fetoprotein (AFP), or squamous cell carcinoma antigen (SCC). Our case may present a new role of CA 19-9 in cases of liver abscess.

## Case Report

An 87-year-old Japanese woman with a 3-day history of a fever of > 38 °C and appetite loss visited at the University of Teikyo Hospital, Japan. At the first visit to our hospital, there were no specific physical findings other than the fever. She was diagnosed with a cold because of symptoms and physical examination results. She started to take acetaminophen. However, her fever and appetite loss remained. She returned 10 days after onset of her symptoms. She was admitted to the hospital for further examination and treatment.

**Figure 1:**
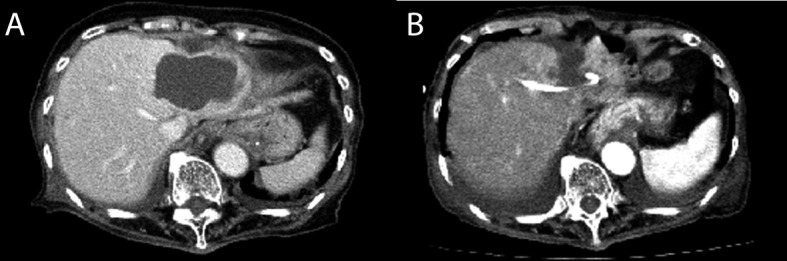
Enhanced liver computed tomography scan on (A) admission and (B) the 3rd day, after the initiation of percutaneous abscess drainage.

On admission, her physical exam revealed a temperature of 38.5 °C, a pulse rate of 92 bpm, and a blood pressure of 130/72 mmHg. Breath sounds were not attenuated, and neither rales nor murmurs were heard. There were no abnormalities on neurological examination, such as neck stiffness or disorientation. Laboratory findings on admission showed a white blood cell count of 10,800/µL with a shift to the left, a C-reactive protein level of 12.09 mg/dL, AST level of 20 U/L, ALT level of 21 IU/L, total bilirubin level of 0.57 mg/dL, amylase level of 41 U/L, creatinine level of 0.70 mg/dL, CEA level of 2.6 ng/mL, AFP level of 1.0 ng/mL, SCC level of 1.1 ng/mL, and CA 19-9 level of 851.0 U/mL. Her admission chest and abdominal X-ray films revealed no significant findings. The chest/abdominal CT revealed a 5 х 6 cm liver abscess on the left lobe (Fig. 1A). Multiple blood cultures were performed.

On the 3rd day after admission, percutaneous liver abscess drainage was initiated (Fig. 1B). Analysis of the fluid revealed pus with a white blood cell count of 769,600/µL and a CA 19-9 level of 13,300 IU/L. After initiation of drainage, piperacillin-tazobactam (4.5 g every 6 hours) was administered. *Escherichia coli* was cultured in both the blood and pus on the 5th day. A liver abscess caused by *E. coli* was diagnosed. After the initial administration of an antibiotic agent and the initiation of liver abscess drainage, the fever rapidly declined. WBC counts decreased and CRP levels declined gradually.

On the 6th day, piperacillin-tazobactam was changed to ampicillin-sulbactam (1.5 g every 6 hours) according to the susceptibility results. Her symptoms also continued to improve. On the 30th day, drainage of the liver abscess was terminated because discharge was no longer present. Intravenous administration of ampicillin-sulbactam was switched to oral administration of levofloxacin (500 mg/day) on the same day. On the 65th day, the liver abscess had disappeared, and treatment with antibiotics was discontinued. This patient had a normal serum CRP level and a serum CA 19-9 level of 46.7 IU/L. This patient was well for 6 months after discontinuing antibiotic treatment.

## Discussion

In our case, the serological level of CA 19-9 was significantly elevated. An extremely high serum CA 19-9 level has occasionally been described in acute cholangitis [5]. However, few patients with benign diseases and a CA 19-9 of > 70 U/mL have been reported [4]. Other reports have shown that high elevations of CA 19-9 (> 40 U/mL) are also rare in patients with benign diseases, although CA 19-9 can be elevated in various benign diseases [5, 6]. There are only two documented cases of liver abscesses associated with a high CA 19-9 level [7, 8]. Our report of this patient, who had a significantly high serum CA 19-9 level associated with a liver abscess, is thought to be very rare. However, detection of CA 19-9 is not usually carried out in cases of liver abscesses. It is possible that high serum CA 19-9 levels can be found in some cases of liver abscesses.

The most common source of pyogenic liver abscesses is the biliary tree, accounting for 40% to 60% of cases. The two major mechanisms for the development of pyogenic liver abscesses are local spread from contiguous infections within the biliary tree or peritoneal cavity and hematogenous seeding [9]. Biliary epithelial cells have a known ability to produce CA 19-9 constitutively, and inflammation can induce increased production of CA 19-9 from the biliary epithelium [10]. Considering these features, liver abscesses, especially transbiliary ones, can easily lead to an inflammatory response of the biliary epithelial cells that are shed into the abscess cavity. Consequently, the tumor marker CA 19-9 is produced in the abscess cavity. Finally, the serum CA 19-9 level may be elevated [10]. This may be the reason why CA 19-9 was significantly increased in our case. In contrast, other tumor markers were not elevated in our case. Elevation of only CA 19-9 among tumor markers might be reasonable in transbiliary liver abscess.

The serum CA 19-9 level was decreased with treatment in our case and in one previously reported case [8]. This outcome is thought to be reasonable because elevation of the serological level of CA 19-9 results from an inflammatory response of the biliary epithelium. Although more investigation is required to further determine the association between liver abscess formation and the serum CA 19-9 level, these results show that CA 19-9 might be a useful marker of treatment response in some cases of liver abscesses.

In conclusion, high serum CA 19-9 levels can be observed in patients with pyogenic liver abscesses. Liver abscess screening in febrile patients with high serological levels of CA 19-9 is a potentially important diagnostic approach for early detection of this infection. Serological CA 19-9 may also serve as a marker of disease progression in cases of liver abscesses.
